# Fibrin clot properties and thrombus composition in cirrhosis

**DOI:** 10.1016/j.rpth.2023.100055

**Published:** 2023-01-20

**Authors:** Ellen G. Driever, Ton Lisman

**Affiliations:** Surgical Research Laboratory, Department of Surgery, University Medical Center Groningen, University of Groningen, Groningen, The Netherlands

**Keywords:** cirrhosis, fibrin, fibrinogen, fibrinolysis, thrombosis

## Abstract

Patients with cirrhosis frequently acquire profound hemostatic alterations, which may affect thrombus quality and composition—factors that determine the susceptibility to embolization and fibrinolysis. In this narrative review, we describe *in vitro* studies on fibrin clot formation and quantitative and qualitative changes in fibrinogen in patients with cirrhosis, and describe recent findings on the composition of portal vein thrombi in patients with cirrhosis. Patients with mild cirrhosis have increased thrombin generation capacity and plasma fibrinogen levels, which may be balanced by delayed fibrin polymerization and decreased factor XIII levels. With progressing illness, plasma fibrinogen levels decrease, but thrombin generation capacity remains elevated. Fibrinogen is susceptible to posttranslational protein modifications and is, for example, hypersialylated and carbonylated in patients with cirrhosis. Despite changes in thrombin generation, factor XIII levels and the fibrinogen molecule, fibrin fiber thickness, and density are normal in patients with cirrhosis. Paradoxically, fibrin clot permeability in patients with cirrhosis is decreased, possibly because of posttranslational protein modifications. Most patients have normal fibrinolytic potential. We have recently demonstrated that portal vein thrombosis is likely a misnomer as the material that may obstruct the cirrhotic portal vein frequently consists of a thickened portal vein wall, rather than a true thrombus. Patients with cirrhosis often have thrombocytopenia and anemia, which may also affect clot stability and composition, but the role of cellular components in clot quality in cirrhosis has not been extensively studied. Finally, we summarize abstracts on fibrin formation and clot quality that were presented at the ISTH 2022 meeting in London.

## Essentials

• Patients with cirrhosis have normal to increased thrombin generation and plasma fibrinogen levels.
• Fibrin formation is delayed and factor XIII levels are decreased in cirrhosis.
• Fibrin fiber thickness and density are normal in cirrhosis, but clot permeability is decreased.
• Fibrin clot quality in acutely ill patients with cirrhosis differs from that of patients with stable cirrhosis.

## 1 Introduction

Patients with liver diseases were historically considered to be at risk of bleeding complications, mainly because of derangements of routine laboratory measures such as prolongation of the prothrombin time, the derived international normalized ratio, and thrombocytopenia. These tests, however, are only sensitive to deficiencies or defects in procoagulant factors, and neglect the simultaneous decline in anticoagulant factors in patients with liver diseases. Indeed, as a result of a reduced capacity of the liver to produce both pro- and anticoagulant factors, patients with liver diseases are in a rebalanced hemostatic state. [1] Rebalanced hemostasis in liver disease has, for example, been demonstrated by a normal to increased thrombin generation capacity using thrombomodulin-modified calibrated automated thrombinography, including the activation of anticoagulant pathways by the addition of thrombomodulin. [[2], [3], [4]]

The net effects of the complex hemostatic alterations in liver diseases have been extensively described. [5] However, there has not been much attention to the quality and composition of thrombi in these patients, but this aspect is of great importance because quality and composition of a thrombus affect mechanical properties, [6] which determine the susceptibility to embolization and fibrinolysis. [7] For example, abnormally dense fibrin structures were found in patients with deep vein thrombosis, coronary artery disease, and stroke, and these dense fibrin clots are more resistant to degradation by plasmin and alter mechanical properties by increasing clot stiffness. [8] In this narrative review, we will describe *in vitro* studies on fibrin clot formation and quantitative and qualitative changes in fibrinogen in patients with liver diseases. In addition, we will discuss changes in coagulation and fibrinolysis and the composition and structure of portal vein thrombi in patients with cirrhosis. Finally, we will shed light on future directions in this field and summarize new data from abstracts on the topic of fibrin clot quality and composition that were presented at the ISTH 2022 annual meeting in London.

## 2 Plasma Fibrinogen Concentration In Patients With Cirrhosis

Fibrinogen is a soluble 340-kD glycoprotein that is primarily synthesized by hepatocytes and is present in the blood of healthy individuals at concentrations between 1.5 and 4 g/L. Fibrinogen levels are normal to increased in patients with stable liver disease. [9] Its levels decrease with increasing severity of disease and may drop below 1.5 g/L in patients with acutely decompensated cirrhosis (AD) and acute-on-chronic liver failure (ACLF). [10] ACLF is a condition that is characterized by critical illness, with disease complications such as ascites, gastrointestinal bleeding, and hepatic encephalopathy, and is associated with multiorgan failure and increased mortality. [11] In the general population, decreased levels of fibrinogen do not necessarily induce a bleeding risk, but are in fact associated with both bleeding and thrombotic complications. [12] In hospitalized patients with liver disease, low fibrinogen levels were a risk factor for bleeding. [13] However, whether there is a causal relationship between hypofibrinogenemia and bleeding in liver disease is unclear. For example, in a study consisting of critically ill patients with cirrhosis, it was showed that administration of cryoprecipitate to correct low (<1.5 g/L) fibrinogen levels did not affect survival or bleeding complications, which suggested that a low fibrinogen level was an additional marker of severity of illness but is not itself a direct factor of bleeding complications in these patients. [14] Indeed, despite low levels of fibrinogen in patients with AD or ACLF, *in vitro*–formed clots from the plasma of these patients have normal to thrombogenic properties. For example, normal clot lysis times and decreased clot permeability (a function of clot pore size) were measured in *in vitro*–formed clots from acutely ill cirrhosis patients, suggesting that factors other than plasma fibrinogen concentration also determine these clot properties. [15] Only when fibrinogen levels were very low (<0.5 U/dL, for example, during liver transplant surgery), and clot stability parameters in *ex vivo* experiments were severely impaired. [16]

## 3 Fibrin Formation In Patients With Cirrhosis

Fibrinogen is converted by thrombin to fibrin, which forms the scaffold of a thrombus. Fibrinogen consists of 2 sets of 3 different polypeptide chains: 2-Aα, 2-Bβ, and 2-γ chains, which are held together by disulfide bridges. [17] It plays a central role in clot formation and stabilization, and is converted to crosslinked fibrin in several steps [18]: 1) proteolytic cleavage of thrombin causes the release of fibrinopeptides and formation of fibrin monomers; 2) linear association of fibrin monomers results in double-stranded protofibrils; 3) association of protofibrils results in formation of fibrin fibers; and 4) factor (F)XIII facilitates covalent crosslinking of polymerized fibrin.

**Figure 1:**
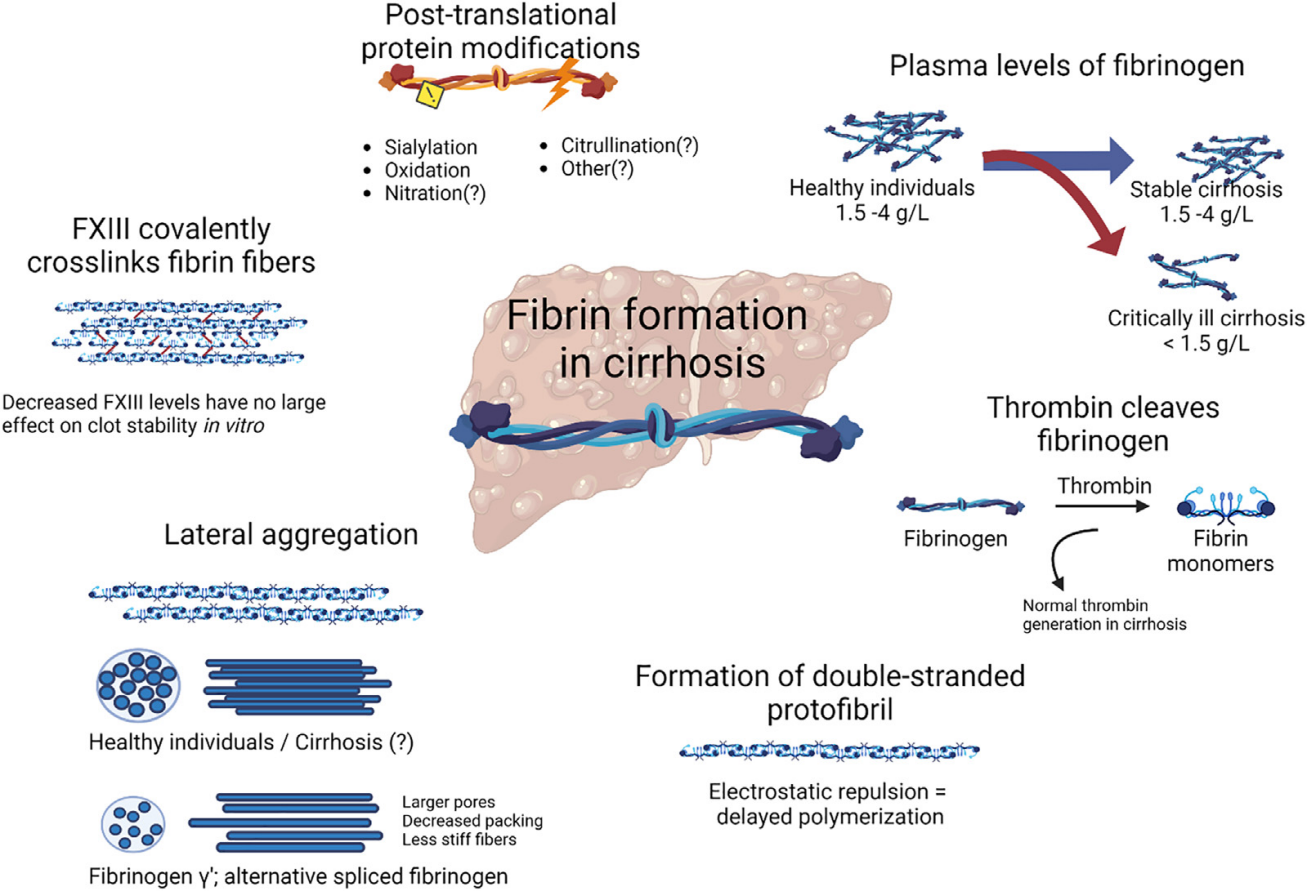
Fibrin formation and fibrinogen properties in patients with cirrhosis. Created with BioRender. FXIII, factor XIII

The process of fibrin clot formation and stabilization and potential changes in the process in patients with liver diseases are outlined in Figure 1 and described hereafter. In the first step, thrombin cleaves fibrinopeptides of the Aα and Bβ chains to produce fibrin monomers. Thrombin levels affect the fibrin clot, with higher levels of thrombin producing a dense network of relatively thin fibrin strands, resulting in less permeable clots and resistance to fibrinolysis. [19,20] Increased thrombin levels have been shown to affect viscoelastic properties of a clot as assessed with thromboelastography (TEG), for example, increased maximum amplitude (which represents the ultimate strength of a fibrin clot) and increased α-angle (which represents the speed of fibrin formation). [21,22] Patients with cirrhosis have normal to increased thrombin generation, [23] which may suggest that levels of thrombin do not largely affect the fibrin clot structure in patients with liver disease. Most stable cirrhosis patients exhibit normal TEG parameters, and hypo- or hypercoagulable TEG profiles were correlated with platelet counts and plasma fibrinogen levels. [24]

The second step of fibrin clot formation is the linear association of fibrin monomers, resulting in double-stranded protofibrils. Fibrin polymerization assays have showed that this step is markedly delayed in patients with liver diseases, with 76% of patients with cirrhosis and 86% of patients with acute liver failure showing abnormal fibrin polymerization rates, [25,26] This delay in polymerization has been explained by increased sialylation of the fibrinogen molecule in patients with liver disease. [27] Sialylation is a posttranslational protein modification, and is a form of glycosylation in which sialic acid is bound at the end of a sugar chain of the protein. It has been suggested that sialic acid inhibits polymerization of fibrin by electrostatic repulsion between fibrin monomers, which impairs the fibrin monomers from interacting with each other. [28] Sialic acid residues are neutralized by calcium, but with increased levels of sialic acid and normal calcium levels, the low-affinity interaction between sialic acid and calcium is inadequate to neutralize the excessive charge repulsion between hypersialylated fibrin molecules, resulting in delayed fibrin polymerization. [28]

In the third step, double-stranded protofibrils are connected to form fibrin fibers. This association can be affected by thrombin concentration and the presence of an alternatively spliced form of fibrinogen, fibrinogen γ′. This form of fibrinogen causes a partially impaired protofibril formation, again likely because of electrostatic repulsion. [29] Higher levels of fibrinogen γ′ have been associated with decreased protofibril packing and less stiff fibrin clots. [30] A study conducted by our group showed normal levels of fibrinogen γ′ in patients with cirrhosis. In addition, similar fibrin fiber density and fiber diameter between cirrhosis patients and healthy individuals were found, suggesting that the intrafibrillar structure is not altered in cirrhosis. [31]

Finally, FXIII, a transglutaminase, is activated by thrombin and becomes FXIIIa, which stabilizes the fibrin clot by forming covalent bonds between fibrin fibers. Thereby, it defines structure, stability, and effector functions of fibrin. [32] Patients with cirrhosis have decreased levels of FXIII, [15] which would theoretically lead to less stable fibrin networks. *In vitro* experiments of fibrin clot structure with plasma from healthy controls and patients with AD and ACLF showed very minor effects on clot parameters after the addition of exogenous FXIII concentrate, despite markedly decreased FXIII levels and reduced clot permeability in patients compared to controls. [15] Nevertheless, it was shown that FXIII does contribute to clot stability in these assays because complete inhibition of FXIIIa activity by T101 (a transglutaminase inhibitor) shortened clot lysis time and lowered permeability in pooled normal plasma. In addition, results of a clot retraction assay (in which isolated platelets and red blood cells [RBCs] from a healthy donor are mixed with patient plasma, coagulation is subsequently initiated with tissue factor and calcium, and the red cell extrusion is measured after 2 hours of clot formation [33]) showed a slightly increased clot weight and a reduced percentage of extruded RBCs in patients compared to controls, despite lower FXIII and fibrinogen levels. Again, full inhibition of FXIII in normal plasma decreased clot weight and increased RBC extrusion, showing that the assay is sensitive for FXIII. In this assay, clot weight is determined by the amount of RBCs that retain within the clot, which also determines the clot size. [34] It could be that only very small amounts of FXIII are necessary for clot protective effects in the assays performed in this study. [15]

## 4 Posttranslational Modifications Of Fibrinogen In Cirrhosis

Acquired dysfibrinogenemia, a term used for altered functionality of the fibrinogen molecule, is common in patients with liver diseases. [35] Dysfibrinogenemia can be a result of posttranslational protein modifications of fibrinogen, of which several are known to affect the function of fibrinogen and therefore affect clot formation and characteristics. [36] Fibrinogen in liver disease is altered compared to healthy individuals with increased sialylation (a form of glycosylation) and oxidation. [[37], [38], [39]]

Hypersialylation leads to delayed fibrin polymerization rates because of electrostatic repulsion between fibrin monomers, as described in the previous section. [28] A recent study performed by our group showed that, despite delayed fibrin polymerization rates in patients with cirrhosis, fibrin clot permeability was decreased, suggesting that although clot formation is delayed, once these clots form they are more thrombogenic compared to healthy controls. [31] Conversely, visual analysis of fibrin clots of glycosylated fibrinogen from healthy donors showed thinner, more-branched fibrin bundles with a more porous network and decreased turbidity, suggesting that glycosylation results in a visually less thrombogenic clot structure. [40] Interestingly, when fibrin clots from cirrhotic patients were visualized with electron microscopy, fiber thickness and density were similar to those of controls. [31] These findings suggest that sialic acid content of fibrin(ogen) in cirrhosis may affect polymerization rates and results in decreased permeability, but the structure of a matured clot may also be affected by mechanisms other than sialylation of fibrinogen alone. For example, carbonylation and nitration of fibrinogen (which may be increased in patients with cirrhosis because of oxidative stress and subsequent production of reactive oxygen and nitrogen species) have distinct effects on clot structure. Nitration of fibrinogen has, for example, been shown in hepatic fibrin deposits in acetaminophen-induced acute liver failure in mice, and was associated with delayed fibrin polymerization rates and reduced clot turbidity. [41] Nitration of normal donor-fibrinogen has been demonstrated to result in large bundles of thin fibrin fibers with large pores between the fibers in 1 study, [42] but showed more thrombogenic clot properties in another study. [43] Carbonylation (a form of oxidation) of fibrinogen has been proposed to increase the thrombogenicity of fibrin clots, for example, in the context of patients with acute myocardial infarction. [44] Nevertheless, conflicting reports on the effects of fibrinogen carbonylation on fibrin structure and function have appeared in the literature. For example, 1 study demonstrated decreased fibrin polymerization with thinner fibers and resistance to lysis in hypercarbonylated fibrinogen from patients with myocardial infarction, [44] whereas another study showed enhanced polymerization in a similar setting. [45] Interestingly, fibrinogen carbonyl content is increased in patients with cirrhosis, and was inversely related to clot permeability. [31] It could be that in patients with cirrhosis, multiple posttranslational protein modifications have opposing effects on clot structure, resulting in a net neutral effect with a clot structure that is indistinguishable from that of controls when examined with electron microscopy or immunofluorescence techniques. Our observations on decreased permeability with a normal clot structure may reflect the complex posttranslational changes in the fibrinogen molecule. For example, in patients with cirrhosis, decreased permeability, which is normally accompanied by alterations in clot architecture, may be a result of increased hydrophobicity of the clot that results in enhanced fluid retention within the clot.

Besides sialylation, nitration, and oxidation of fibrinogen, other posttranslational modifications may also affect fibrinogen in patients with liver diseases. For example, citrullination of proteins by peptidyl arginine deiminase (PAD) enzymes [46] possibly modifies the fibrinogen molecule in patients with liver disease. PAD enzymes, specifically PAD4, play an important role in the formation of neutrophil extracellular traps (NETs), and neutrophil extracellular trap formation have been demonstrated to play a role in progression and complications of liver disease. [47] The presence of citrullinated fibrinogen has for example been shown in pathologies such as rheumatoid arthritis. [48] Citrullination of fibrinogen resulted in thinner fibrin fibers with increased fiber density and lower clot permeability. [49] Citrullination of fibrinogen and its potential consequences in liver diseases should be subject to future research. In addition, as fibrinogen is 1 of the most abundant plasma proteins and is very susceptible to modification, other posttranslational protein modifications, such as inflammation-associated changes in fibrin fiber thickness, [50] may also have an effect on fibrin in patients with liver diseases. Future studies are required to study these changes and investigate the effect on clot properties.

## 5 Fibrinolysis And Permeability Of Fibrin Clots In Cirrhosis

We have studied the stability of clots generated from plasma in patients with chronic liver diseases using 2 distinct assays: a plasma-based clot lysis test and a clot permeability assay. Both tests are sensitive for levels and function of key proteins involved in clot formation and breakdown. [51,52] Historically, patients with cirrhosis were classified as hyperfibrinolytic. [53] A hyperfibrinolytic state in patients with cirrhosis has been linked to decreased levels of antiplasmin and thrombin-activatable fibrinolysis inhibitor or increased levels of tPA, and was described to potentially contribute to bleeding complications in these patients. [54,55] More recent studies used global assays of fibrinolysis, and found a normal fibrinolytic phenotype in patients with compensated or stably decompensated cirrhosis, which was explained by a simultaneous decline in pro- and antifibrinolytic factors. [9,56] Notably, in these studies, individual patients had hypo- or hyperfibrinolytic profiles. [9,56] Others, however, exhibited hyperfibrinolysis on a group level using a similar methodology, who were apparently similar patients. The discrepancies may be explained by differences in methodology and selected patients. [54,[56], [57], [58], [59]] For example, in patients with mild cirrhosis caused by nonalcoholic fatty liver disease or cholestatic liver disease, we demonstrated hypofibrinolytic profiles, which were not present in patients with cirrhosis related to alcohol or viral hepatitis. [9,60] Finally, although patients with stable cirrhosis may have hyperfibrinolytic features based on laboratory measures, hyperfibrinolysis-related bleeding complications in these patients are exceedingly rare. [58]

Whereas patients with relatively stable liver disease mostly have normal fibrinolytic phenotypes, a recent study by our group has showed that patients with AD and ACLF, with higher disease severity and additional disease complications such as presence of ascites or development of hepatic encephalopathy, have very variable fibrinolytic phenotypes. [61] Patients with AD were primarily hyperfibrinolytic and patients with ACLF were primarily hypofibrinolytic. Patients with ACLF and hypofibrinolysis often had sepsis. Indeed, sepsis in patients without an underlying liver disease is often accompanied by a hypofibrinolytic state, which can be explained by high levels of plasminogen activator inhibitor-1 in patients with sepsis. [[61], [62], [63]] Other comorbidities such as diabetes mellitus: type II or use of drugs, such as anticoagulants or statins, also affect fibrinolysis. [64] How these factors affect fibrinolysis in patients with liver disease has not yet been elucidated and should be subject to further research.

Clot permeability, another key measure of clot structure and function, is decreased in patients with cirrhosis when measured using an experimental setup in which permeation is tested by measuring fluid permeation through the clot by the force of gravity. These thrombogenic clot properties were observed despite the delayed fibrinogen to fibrin conversion, and were present even in patients with decreased plasma fibrinogen levels. Notably, decreased permeability was observed despite unaltered fibrin fiber thickness or pore size within the clot. [31] To better understand these paradoxes, we recently reanalyzed fibrin permeation and compared fibrin clot permeability assessed by the force of gravity with permeability assessed by compressional force, where a clot is formed between 2 parallel plates of a rheometer and fluid is pressed out of the fibrin network by lowering the upper plate. [65] We found that under the force of gravity, permeability decreases with increasing severity of the disease. In contrast, when permeability was assessed using rheometry, no differences in permeability were observed between patients and controls (unpublished data). Ongoing studies are focused on addressing the reason for this discrepancy. We hypothesize that the studies under the force of gravity may show decreased permeability not because the clot is truly more thrombogenic but because the increased negative charge in the clot retains water. In studies under compressional force, the electrostatic repulsion may not be strong enough to retain water in the fibrin network.

## 6 Clot Composition In Cirrhosis

The major determinant of clot stability and quality is fibrin, and the effects of alterations in fibrinogen and fibrin have been extensively studied in patients with liver diseases as discussed in the previous sections. Other components of the thrombus, such as platelets and RBCs, also contribute to clot stability and quality, but these have not been studied in patients with liver diseases yet. Patients with liver diseases are often diagnosed with thrombocytopenia, anemia, and changes in white blood cell counts and function, [66] and these alterations may affect thrombus composition and mechanical characteristics. [67,68] The composition of venous and arterial thrombi has been extensively studied in the general population, [69] but data from patients with liver diseases are not available yet.

Venous thrombi consist mainly of RBCs and fibrin. [69] Upon clot contraction of venous thrombi, a phenomenon driven by activated platelets, RBCs undergo deformation and become polyhedral structures. [70] Contraction of venous thrombi causes volume shrinkage of the thrombus, and determines the degree of vessel obstruction and the likelihood of thrombus mechanical rupture, which may lead to thrombotic embolization. [71,72] The majority of RBCs within a thrombus are of polyhedral shape and are called polyhedrocytes. [70]

**Figure 2:**
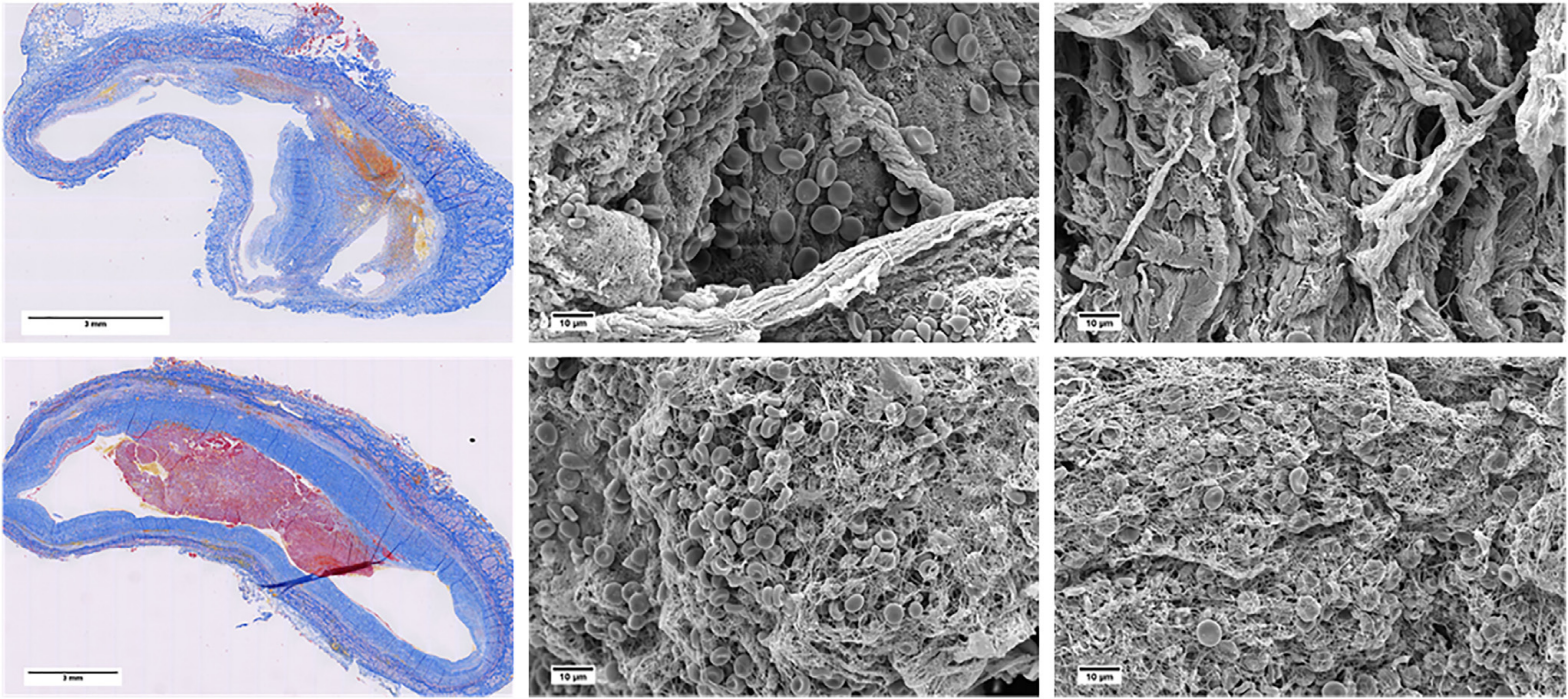
Composition of nonmalignant portal vein thrombi in patients with cirrhosis. Top panels: MSB-stained section (left) of an extrahepatic portal vein thrombus showing intimal hyperplasia with some hemorrhage, but no fibrin. The scanning electron microscopy images of the same thrombus sample (middle and right images of the upper panel ) show collagen bundles, but no fibrin and few erythrocytes. Bottom panel: MSB-stained section (left) of a portal vein thrombus showing intimal hyperplasia with a fibrin-rich thrombus within the lumen of the vessel. The scanning electron microscopy images (middle and right images of the lower panel) show a fibrin mesh with erythrocytes entrapped. Note that most erythrocytes are in their biconcave shape and not in a polyhedral shape that is often observed in contracted blood clots or thrombi. MSB, Martius Scarlet Blue

Recently, we studied the composition of portal vein thrombi in patients with cirrhosis who underwent liver transplantation, [73] and observed RBCs to be of biconcave shape instead of their polyhedral shape in those thrombi (Figure 2). [71,74] This interesting and unexpected finding suggests that the portal vein thrombus in cirrhosis does not have the same features as other venous thrombi. Future studies should investigate whether platelet activation differs in these thrombi or whether RBCs in cirrhotic portal vein thrombi are unable to deform into a polyhedral shape, for example, because of changes in the lipid composition of the red cell membrane. [75,76]

Moreover, in this study on the structure and composition of cirrhotic portal vein thrombi, we found that the thrombi lacked a fibrin-rich part in two-thirds of the cases, whereas all thrombi consisted of a thickened, fibrotic vessel wall (Figure 2). In other words, portal vein thrombi often lack “classical” thrombus components that are uniformly present in thrombi isolated from patients with deep vein thrombosis, pulmonary embolism, myocardial infarction, and stroke. [69,7] Rather, portal vein thrombi appear to consist of intimal fibrosis, which in in some cases were overlaid by a fibrin-rich clot. These observations suggest that the term “portal vein thrombosis” may be a misnomer and that the terms “portal vein stenosis” or “nonmalignant portal vein occlusion” may be more appropriate. We propose 2 possible mechanisms by which portal vein intimal fibrosis develops: 1) an initial fibrin-rich thrombus organizes into a fibrotic structure that re-endothelializes over time, and/or 2) intimal fibrosis develops in the absence of overt initial fibrin formation and is a result of, for example, portal hypertension and vascular endothelial cell stress. These findings may also explain why portal vein thrombi do not always recanalize after treatment with anticoagulant therapy. The current pharmacologic treatment of portal vein thrombosis consists of low-molecular-weight heparins or direct oral anticoagulants, but this therapy does not always lead to recanalization of the portal vein. [77] Treatment with anticoagulants seems to be most effective in more recently formed thrombi, which may consist of fibrin. A thrombus that only contains fibrotic tissue and no fibrin does not likely respond to anticoagulants. Future studies should specifically focus on the pathophysiology and the prevention and treatment of portal vein intimal hyperplasia.

## 7 Conclusions And Future Directions

In summary, the coagulation system of patients with compensated or stably decompensated cirrhosis is in a rebalanced state, characterized by normal to increased thrombin generation and plasma fibrinogen levels. Increased thrombin generation capacity and fibrinogen levels may be balanced by delayed fibrin formation and decreased FXIII levels compared to healthy subjects. Interestingly, despite changes in the fibrinogen molecule, FXIII levels, and thrombin generation, fibrin fiber density and thickness are normal in patients with cirrhosis. Paradoxically, the permeability of clots formed with plasma from patients with cirrhosis is decreased, suggesting a more thrombogenic structure. These findings may be explained by posttranslational protein modifications of fibrinogen in liver disease, notably oxidation. Interestingly, when permeability was measured under compression, no differences were found between patients and controls, suggesting that the permeation under gravity assay overestimates thrombogenic capacity of clots from patients with cirrhosis, possibly because of retention of water as a result of hypersialylation.

It would be interesting to study the deformational changes in the fibrin clots under compressional forces in more detail, for example, by analyzing clot stiffness in between subsequent compression steps or by analysis with scanning electron microscopy. Such experiments would give more insights into the strength and stability of the fibrin fibers.

Fibrin clot quality in patients with AD or ACLF may be different from that of patients with stable cirrhosis, as fibrinogen and FXIII levels are substantially decreased in these patients. Indeed, fibrinolysis is increased in patients with AD and markedly impaired in patients with ACLF, which was ascribed to increased levels of plasminogen activator inhibitor-1. Other parameters of fibrin clot formation and fibrin clot quality could also be altered in critically ill patients with cirrhosis. For example, systemic inflammation in these patients causes alterations in the hemostatic state, [78] may increase fibrinogen protein modifications, and affect fibrin polymerization rates and permeability of fibrin clots.

Future studies on thrombus quality and stability in patients with liver diseases should include effects of platelets and RBCs. Patients with liver diseases are thrombocytopenic and often have anemia, which are factors that affect clot characteristics. [66,68] In addition, our recent findings on portal vein thrombi composition and structure raised several questions. For example, future studies should investigate clot contraction and deformation of RBCs to polyhedrocytes in cirrhotic portal vein thrombosis and other thrombotic complications in patients with cirrhotic. Further research is also required to gain more knowledge about the pathophysiology of portal vein thrombosis in cirrhosis, as we found that it consists of fibrin-rich thrombi in only one-third of the cases, whereas all cases had intimal thickening of the vessel wall. More research is required to provide more rational preventive and treatment strategies in patients with cirrhosis and portal vein thrombosis.

Taken together, patients with cirrhosis have unique alterations in fibrinogen concentration, posttranslational modification of the fibrinogen molecule, and decreased FXIII concentration. Despite these extensive alterations, clot formation appears remarkably preserved, which may relate to prothrombotic posttranslational modifications such as oxidation. These insights may help interpret findings of hypofibrinogenemia in cirrhosis and question liberal infusion of the fibrinogen concentrate. Clinical studies are required to address situations in which fibrinogen concentrate may be beneficial for these patients.

## 8 ISTH London 2022 Report

A number of abstracts on clot quality and composition were presented at the ISTH 2022 congress in London. In this section, we will briefly summarize studies on the topic of fibrin clot stability and composition.

Luyendyk et al. used murine models to investigate intrahepatic microthrombosis in acetaminophen-induced acute liver injury [79]. An abstract that was presented at the ISTH 2022 by Poole et al. [81] describes the effects of acetaminophen overdose in mice with a mutation in the fibrinogen γ-chain, making fibrin(ogen) incapable of interacting with platelet integrin αIIbβ3 (*FibγΔ5)*. It was previously showed that acetaminophen overdose causes accumulation of fibrinogen and platelets in the injured liver, [79,80] but the mechanisms initiating platelet accumulation in the injured liver are not understood. Poole et al. [81] hypothesized that hepatic platelet accumulation in the acetaminophen-injured liver is mediated by fibrin(ogen) engagement of the platelet integrin αIIbβ3. Surprisingly, *FibγΔ5* mice had modestly higher platelet accumulation than wild-type mice after acetaminophen challenge. In addition, the *FibγΔ5* mice had enhanced hepatic accumulation of high-molecular-weight crosslinked γ-chain multimers of fibrin, but no γ-γ dimer formation. Fibrin clots from the mutant mice were denser and consisted of thinner fibers compared with the clots from wild-type mice. The effect was independent of platelet integrin αIIbβ3-fibrinogen interactions, as treatment of wild-type mice with an αIIbβ3 inhibitor had no effect on acetaminophen-induced hepatic necrosis. The aberrant fibrinogen crosslinking may exacerbate liver injury following acetaminophen overdose.

More abstracts studied crosslinking of α- and γ-chains of fibrin. These crosslinking events increase clot stiffness and stabilize the final clot. In the study by Feller et al. [82] presented at the ISTH 2022, they described a mouse model in which α-α crosslinking was impaired by the introduction of 4-point mutations in the fibrinogen α-chain. Clots made of plasma obtained from these mice rupture at lower stress and have reduced toughness compared with clots made of plasma from wild-type mice. In another abstract, this research group [83] described their study on the impact of impaired α-crosslinking on venous thromboembolism in the mouse model. The clotting time was delayed, clot firmness and contraction were reduced, and the mutant mice was at a higher risk of embolization. In a previous study, the authors described that in the absence of γ-chain crosslinking, [84] fibrin fibers rupture at lower stress and have reduced toughness, resulting in more frequent embolization of clots in a mouse model of vena cava thrombosis. Based on the results from these studies, the investigators concluded that deficiency in both α- and γ-chain crosslinking results in fibers that are more prone to rupture and that α- and γ-crosslinking play complementary roles in generating key biochemical properties of fibrin clots for the prevention of embolism.

A study presented by Fish et al. [85] showed the effects of fibrinogen γ-chain knockdown in larval zebrafish and of a large deletion in the zebrafish fibrinogen γ-gene. Knockdown of fibrinogen γ-chain expression prevented laser-induced venous thrombosis, which was assessed by measuring the time until occlusion of the vessel. The authors also generated zebrafish with a deletion in the fibrinogen γ-gene, which showed no blood coagulation or thrombosis after laser-induced injury. The authors proposed that this model can serve as a stable knockout background to study the effects of specific mutations of fibrinogen γ-chain expression.

Several studies described fibrin clot properties such as permeability and susceptibility to lysis in *in vitro* experimental setups. For example, Klajmon et al. [86] described reduced clot permeability and prolonged clot lysis times in fibrin clots made of plasma from patients with genetically confirmed antithrombin deficiency. The findings suggest a mechanism by which antithrombin deficiency contributes to thrombosis risk. Another study by Smith and Morrissey [87] described that fibrinogen that was incubated with a neutrophil-released enzyme (cathepsin G, for example, released during inflammatory responses) causes cleavage of fibrinogen, which results in faster polymerization rates of fibrin induced by thrombin, higher clot turbidity, weaker clots, and decreased lysis times compared to control. The authors concluded that release of cathepsin G from activated neutrophils may affect clot formation in the presence of an ongoing inflammatory response, with potential consequences for thrombosis.

Another interesting abstract was presented by Risman et al. [88] about clot contraction and its impact on fibrinolysis. By using a combination of mathematical modeling and experimental methodologies to characterize the process of external fibrinolysis, the authors aimed to understand how key structural changes mechanistically drive the decrease in fibrinolysis of contracted blood clots. In their modeling and experimental approach, the densification of fibrin was the most significant determinant of the rate of fibrinolysis, which the authors ascribed to reduced diffusion of tPA in the clot. These findings may potentially be used therapeutically to optimize timing and delivery of lytic agents.

## References

[1] Lisman T., Porte R.J. (2010). Rebalanced hemostasis in patients with liver disease: evidence and clinical consequences. Blood.

[2] Lisman T., Bakhtiari K., Adelmeijer J., Meijers J.C.M.C.M., Porte R.J.J., Stravitz R.T.T. (2012). Intact thrombin generation and decreased fibrinolytic capacity in patients with acute liver injury or acute liver failure. J Thromb Haemost.

[3] Potze W., Porte R.J., Lisman T. (2015). Management of coagulation abnormalities in liver disease. Expert Rev Gastroenterol Hepatol.

[4] Tripodi A., Salerno F., Chantarangkul V., Clerici M., Cazzaniga M., Primignani M. (2005). Evidence of normal thrombin generation in cirrhosis despite abnormal conventional coagulation tests. Hepatology.

[5] Lisman T., Porte R.J. (2017). Pathogenesis, prevention, and management of bleeding and thrombosis in patients with liver diseases. Res Pract Thromb Haemost.

[6] Brass L.F., Diamond S.L. (2016). Transport physics and biorheology in the setting of hemostasis and thrombosis. J Thromb Haemost.

[7] Alkarithi G., Duval C., Shi Y., Macrae F.L., Ariëns R.A.S. (2021). Thrombus structural composition in cardiovascular disease. Arterioscler Thromb Vasc Biol.

[8] Ariëns R.A.S. (2016). Novel mechanisms that regulate clot structure/function. Thromb Res.

[9] Bos S., van den Boom B., Kamphuisen P.W., Adelmeijer J., Blokzijl H., Schreuder T. (2019). Haemostatic profiles are similar across all aetiologies of cirrhosis. Thromb Haemost.

[10] Fisher C., Patel V.C., Stoy S.H., Singanayagam A., Adelmeijer J., Wendon J. (2018). Balanced haemostasis with both hypo- and hyper-coagulable features in critically ill patients with acute-on-chronic-liver failure. J Crit Care.

[11] Jalan R., Gines P., Olson J.C., Mookerjee R.P., Moreau R., Garcia-Tsao G. (2012). Acute-on chronic liver failure. J Hepatol.

[12] Casini A., de Moerloose P., Neerman-Arbez M. (2016). Clinical features and management of congenital fibrinogen deficiencies. Semin Thromb Hemost.

[13] Drolz A., Horvatits T., Roedl K., Rutter K., Staufer K., Kneidinger N. (2016). Coagulation parameters and major bleeding in critically ill patients with cirrhosis. Hepatology.

[14] Budnick I.M.M., Davis J.P.E., Sundararaghavan A., Konkol S.B.B., Lau C.E.E., Alsobrooks J.P.P. (2021). Transfusion with cryoprecipitate for very low fibrinogen levels does not affect bleeding or survival in critically ill cirrhosis patients. Thromb Haemost.

[15] Blasi A., Patel V.C., Spanke E.N.H.E., Adelmeijer J., Stamouli M., Zamalloa A. (2022). Fibrin clot quality in acutely ill cirrhosis patients: relation with outcome and improvement with coagulation factor concentrates. Liver Int.

[16] Groeneveld D.J., Adelmeijer J., Hugenholtz G.C.G., Ariëns R.A.S., Porte R.J., Lisman T. (2015). Ex vivo addition of fibrinogen concentrate improves the fibrin network structure in plasma samples taken during liver transplantation. J Thromb Haemost.

[17] Neerman-Arbez M., de Moerloose P., Casini A. (2016). Laboratory and genetic investigation of mutations accounting for congenital fibrinogen disorders. Semin Thromb Hemost.

[18] Litvinov R.I., Pieters M., de Lange-Loots Z., Weisel J.W. (2021). Fibrinogen and fibrin. Subcell Biochem.

[19] Blombäck B., Carlsson K., Hessel B., Liljeborg A., Procyk R., Aslund N. (1989). Native fibrin gel networks observed by 3D microscopy, permeation and turbidity. Biochim Biophys Acta.

[20] Blombäck B., Carlsson K., Fatah K., Hessel B., Procyk R. (1994). Fibrin in human plasma: gel architectures governed by rate and nature of fibrinogen activation. Thromb Res.

[21] Wolberg A.S., Campbell R.A. (2008). Thrombin generation, fibrin clot formation and hemostasis. Transfus Apher Sci.

[22] Zeng Z., Fagnon M., Nallan Chakravarthula T., Alves N.J. (2020). Fibrin clot formation under diverse clotting conditions: comparing turbidimetry and thromboelastography. Thromb Res.

[23] Groeneveld D., Porte R.J., Lisman T. (2014). Thrombomodulin-modified thrombin generation testing detects a hypercoagulable state in patients with cirrhosis regardless of the exact experimental conditions. Thromb Res.

[24] Hugenholtz G.C.G.C.G., Lisman T., Stravitz R.T.T. (2017). Thromboelastography does not predict outcome in different etiologies of cirrhosis. Res Pract Thromb Haemost.

[25] Palascak J.E., Martinez J. (1977). Dysfibrinogenemia associated with liver disease. J Clin Invest.

[26] Francis J.L., Armstrong D.J. (1982). Acquired dysfibrinogenaemia in liver disease. J Clin Pathol.

[27] Martinez J., Palascak J.E., Kwasniak D. (1978). Abnormal sialic acid content of the dysfibrinogenemia associated with liver disease. J Clin Invest.

[28] Dang C.V., Shin C.K., Bell W.R., Nagaswami C., Weisel J.W. (1989). Fibrinogen sialic acid residues are low affinity calcium-binding sites that influence fibrin assembly. J Biol Chem.

[29] Weisel J.W., Litvinov R.I. (2017). Fibrin formation, structure and properties. Subcell Biochem.

[30] Macrae F.L., Domingues M.M., Casini A., Ariëns R.A.S. (2016). The (patho)physiology of fibrinogen γ. Semin Thromb Hemost.

[31] Hugenholtz G.C.G.C.G., Macrae F., Adelmeijer J., Dulfer S., Porte R.J.J., Lisman T. (2016). Procoagulant changes in fibrin clot structure in patients with cirrhosis are associated with oxidative modifications of fibrinogen. J Thromb Haemost.

[32] Kattula S., Byrnes J.R.R., Wolberg A.S.S. (2017). Fibrinogen and fibrin in hemostasis and thrombosis. Arterioscler Thromb Vasc Biol.

[33] Kattula S., Byrnes J.R., Martin S.M., Holle L.A., Cooley B.C., Flick M.J. (2018). Factor XIII in plasma, but not in platelets, mediates red blood cell retention in clots and venous thrombus size in mice. Blood Adv.

[34] Aleman M.M., Byrnes J.R., Wang J.G., Tran R., Lam W.A., di Paola J. (2014). Factor XIII activity mediates red blood cell retention in venous thrombi. J Clin Invest.

[35] May J.E., Wolberg A.S., Lim M.Y. (2021). Disorders of fibrinogen and fibrinolysis. Hematol Oncol Clin North Am.

[36] de Vries J.J., Snoek C.J.M., Rijken D.C., de Maat M.P.M. (2020). Effects of post-translational modifications of fibrinogen on clot formation, clot structure, and fibrinolysis: a systematic review. Arterioscler Thromb Vasc Biol.

[37] Nagel T., Klaus F., Ibanez I.G., Wege H., Lohse A., Meyer B. (2018). Fast and facile analysis of glycosylation and phosphorylation of fibrinogen from human plasma—correlation with liver cancer and liver cirrhosis. Anal Bioanal Chem.

[38] Martinez J., Keane P.M., Gilman P.B., Palascak J.E. (1983). The abnormal carbohydrate composition of the dysfibrinogenemia associated with liver disease. Ann N Y Acad Sci.

[39] Lisman T., Ariëns R.A.S. (2016). Alterations in fibrin structure in patients with liver diseases. Semin Thromb Hemost.

[40] Langer B.G., Weisel J.W., Dinauer P.A., Nagaswami C., Bell W.R. (1988). Deglycosylation of fibrinogen accelerates polymerization and increases lateral aggregation of fibrin fibers. J Biol Chem.

[41] Poole L.G., Kopec A.K., Groeneveld D.J., Pant A., Baker K.S., Cline-Fedewa H.M. (2021). Factor XIII cross-links fibrin(ogen) independent of fibrin polymerization in experimental acute liver injury. Blood.

[42] Vadseth C., Souza J.M., Thomson L., Seagraves A., Nagaswami C., Scheiner T. (2004). Pro-thrombotic state induced by post-translational modification of fibrinogen by reactive nitrogen species. J Biol Chem.

[43] Parastatidis I., Thomson L., Burke A., Chernysh I., Nagaswami C., Visser J. (2008). Fibrinogen β-chain tyrosine nitration is a prothrombotic risk factor. J Biol Chem.

[44] Becatti M., Marcucci R., Bruschi G., Taddei N., Bani D., Gori A.M. (2014). Oxidative modification of fibrinogen is associated with altered function and structure in the subacute phase of myocardial infarction. Arterioscler Thromb Vasc Biol.

[45] Paton L.N., Mocatta T.J., Richards A.M., Winterbourn C.C. (2010). Increased thrombin-induced polymerization of fibrinogen associated with high protein carbonyl levels in plasma from patients post myocardial infarction. Free Radic Biol Med.

[46] Liu X., Arfman T., Wichapong K., Reutelingsperger C.P.M., Voorberg J., Nicolaes G.A.F. (2021). PAD4 takes charge during neutrophil activation: impact of PAD4 mediated NET formation on immune-mediated disease. J Thromb Haemost.

[47] Meijenfeldt F.A.V., Jenne C.N. (2020). Netting liver disease: neutrophil extracellular traps in the initiation and exacerbation of liver pathology. Semin Thromb Hemost.

[48] Masson-Bessière C., Sebbag M., Girbal-Neuhauser E., Nogueira L., Vincent C., Senshu T. (2001). The major synovial targets of the rheumatoid arthritis-specific antifilaggrin autoantibodies are deiminated forms of the α- and β-chains of fibrin. J Immunol.

[49] Varjú I., Tóth E., Farkas Á.Z., Farkas V.J., Komorowicz E., Feller T. (2022). Citrullinated fibrinogen forms densely packed clots with decreased permeability. J Thromb Haemost.

[50] Li R., Ren M., Luo M., Chen N., Zhang Z., Luo B. (2012). Monomeric C-reactive protein alters fibrin clot properties on endothelial cells. Thromb Res.

[51] Bryk A.H., Natorska J., Ząbczyk M., Zettl K., Wiśniewski J.R., Undas A. (2020). Plasma fibrin clot proteomics in patients with acute pulmonary embolism: association with clot properties. J Proteomics.

[52] Ząbczyk M., Stachowicz A., Natorska J., Olszanecki R., Wiśniewski J.R., Undas A. (2019). Plasma fibrin clot proteomics in healthy subjects: relation to clot permeability and lysis time. J Proteomics.

[53] Goodpasture E.W. (1914). Fibrinolysis in chronic hepatic insufficiency. Bull Johns Hopkins Hosp.

[54] Colucci M., Binetti B.M., Branca M.G., Clerici C., Morelli A., Semeraro N. (2003). Deficiency of thrombin activatable fibrinolysis inhibitor in cirrhosis is associated with increased plasma fibrinolysis. Hepatology.

[55] Leebeek F.W., Kluft C., Knot E.A., de Maat M.P., Wilson J.H. (1991). A shift in balance between profibrinolytic and antifibrinolytic factors causes enhanced fibrinolysis in cirrhosis. Gastroenterology.

[56] Lisman T., Leebeek F.W.G., Mosnier L.O., Bouma B.N., Meijers J.C.M., Janssen H.L.A. (2001). Thrombin-activatable fibrinolysis inhibitor deficiency in cirrhosis is not associated with increased plasma fibrinolysis. Gastroenterology.

[57] Leebeek F.W.G., Rijken D.C. (2015). The fibrinolytic status in liver diseases. Semin Thromb Hemost.

[58] von Meijenfeldt F.A.A., Lisman T. (2021). Fibrinolysis in patients with liver disease. Semin Thromb Hemost.

[59] Rijken D.C., Kock E.L., Guimarães A.H.C., Talens S., Darwish Murad S., Janssen H.L.A. (2012). Evidence for an enhanced fibrinolytic capacity in cirrhosis as measured with two different global fibrinolysis tests. J Thromb Haemost.

[60] Potze W., Siddiqui M.S., Boyett S.L., Adelmeijer J., Daita K., Sanyal A.J. (2016). Preserved hemostatic status in patients with non-alcoholic fatty liver disease. J Hepatol.

[61] Blasi A., Patel V.C.C., Adelmeijer J., Azarian S., Hernandez Tejero M., Calvo A. (2020). Mixed fibrinolytic phenotypes in decompensated cirrhosis and acute-on-chronic liver failure with hypofibrinolysis in those with complications and poor survival. Hepatology.

[62] Levi M., van der Poll T. (2017). Coagulation and sepsis. Thromb Res.

[63] Siudut J., Natorska J., Wypasek E., Wiewiórka Ł., Ostrowska-Kaim E., Wiśniowska-Śmiałek S. (2020). Impaired fibrinolysis in patients with isolated aortic stenosis is associated with enhanced oxidative stress. J Clin Med.

[64] Undas A., Ariëns R.A.S. (2011). Fibrin clot structure and function: a role in the pathophysiology of arterial and venous thromboembolic diseases. Arterioscler Thromb Vasc Biol.

[65] Punter M.T.J.J.M., Vos B.E., Mulder B.M., Koenderink G.H. (2020). Poroelasticity of (bio)polymer networks during compression: theory and experiment. Soft Matter.

[66] Qamar A.A., Grace N.D. (2009). Abnormal hematological indices in cirrhosis. Can J Gastroenterol.

[67] Roeloffzen W.W., Kluin-Nelemans H.C., Mulder A.B., de Wolf J.T. (2010). Thrombocytopenia affects plasmatic coagulation as measured by thrombelastography. Blood Coagul Fibrinolysis.

[68] Byrnes J.R., Wolberg A.S. (2017). Red blood cells in thrombosis. Blood.

[69] Chernysh I.N., Nagaswami C., Kosolapova S., Peshkova A.D., Cuker A., Cines D.B. (2020). The distinctive structure and composition of arterial and venous thrombi and pulmonary emboli. Sci Rep.

[70] Tutwiler V., Mukhitov A.R., Peshkova A.D., le Minh G., Khismatullin R.R., Vicksman J. (2018). Shape changes of erythrocytes during blood clot contraction and the structure of polyhedrocytes. Sci Rep.

[71] Peshkova A.D., Malyasyov D.V., Bredikhin R.A., le Minh G., Andrianova I.A., Tutwiler V. (2018). Reduced contraction of blood clots in venous thromboembolism is a potential thrombogenic and embologenic mechanism. TH Open.

[72] Tutwiler V., Peshkova A.D., le Minh G., Zaitsev S., Litvinov R.I., Cines D.B. (2019). Blood clot contraction differentially modulates internal and external fibrinolysis. J Thromb Haemost.

[73] Driever E.G., von Meijenfeldt F.A., Adelmeijer J., de Haas R.J., van den Heuvel M.C., Nagasami C. (2022). Non-malignant portal vein thrombi in patients with cirrhosis consist of intimal fibrosis with or without a fibrin-rich thrombus. Hepatology.

[74] Zalewski J., Lewicki L., Krawczyk K., Zabczyk M., Targonski R., Molek P. (2019). Polyhedral erythrocytes in intracoronary thrombus and their association with reperfusion in myocardial infarction. Clin Res Cardiol.

[75] Salvioli G., Rioli G., Lugli R., Salati R. (1978). Membrane lipid composition of red blood cells in liver disease: regression of spur cell anaemia after infusion of polyunsaturated phosphatidylcholine. Gut.

[76] Owen J.S., Bruckdorfer K.R., Day R.C., McIntyre N. (1982). Decreased erythrocyte membrane fluidity and altered lipid composition in human liver disease. J Lipid Res.

[77] Northup P.G., Davis J.P.E. (2018). Timing of anticoagulation for portal vein thrombosis in liver cirrhosis: a US hepatologist’s perspective. J Transl Int Med.

[78] Driever E.G., Lisman T. (2022). Effects of inflammation on hemostasis in acutely ill patients with liver disease. Semin Thromb Hemost.

[79] Groeneveld D., Cline-Fedewa H., Baker K.S., Williams K.J., Roth R.A., Mittermeier K. (2020). Von Willebrand factor delays liver repair after acetaminophen-induced acute liver injury in mice. J Hepatol.

[80] Ganey P.E., Luyendyk J.P., Newport S.W., Eagle T.M., Maddox J.F., Mackman N. (2007). Role of the coagulation system in acetaminophen-induced hepatotoxicity in mice. Hepatology.

[81] Poole L., Groeneveld D., Cline-Fedewa H., Flick M., Luyendyk J. (2022). Dysregulated fibrinogen γ-chain cross-linking in FibγΔ5 mice drives acute liver injury after acetaminophen overdose [Abstract]. Res Pract Thromb Haemost.

[82] Feller T., Duval C., Connell S., Ariëns R. (2022). Both α- and γ-chain crosslinks mediated by FXIIIa affect fibrin fibre resistance to rupture. Res Pract Thromb Haemost.

[83] Duval C., Feller T., McPherson H., Ariëns R. (2022). A new mouse model of impaired fibrin α-chain crosslinking shows increased venous thromboembolism [Abstract]. Res Pract Thromb Haemost.

[84] Duval C., Baranauskas A., Feller T., Ali M., Cheah L.T., Yuldasheva N.Y. (2021). Elimination of fibrin γ-chain cross-linking by FXIIIa increases pulmonary embolism arising from murine inferior vena cava thrombi. Proc Natl Acad Sci U S A.

[85] Fish R., Richard M., Neerman-Arbez M. (2022). A zebrafish model of dysfibrinogenemia caused by hotspot mutations in the human fibrinogen gamma chain [Abstract]. Res Pract Thromb Haemost.

[86] Klajmon A., Kopytek M., Natorska J., Undas A., Ząbczyk M. (2022). Antithrombin deficiency is associated with more compact plasma fibrin clot structure and impaired susceptibility to fibrinolysis [Abstract]. Res Pract Thromb Haemost.

[87] Smith S., Morrissey J. (2022). Neutrophil cathepsin G cleaves fibrinogen affecting clot structure [Abstract]. Res Pract Thromb Haemost.

[88] Risman R., Abdelhamid A., Weisel J., Bannish B., Tutwiler V. (2022). Effects of clot contraction on clot degradation: a mathematical and experimental approach [Abstract]. Res Pract Thromb Haemost.

